# Application of Spectral Phasor analysis to sodium microenvironments in myoblast progenitor cells

**DOI:** 10.1371/journal.pone.0204611

**Published:** 2018-10-31

**Authors:** Hamid Sediqi, Alex Wray, Christopher Jones, Mark Jones

**Affiliations:** School of Science and Health, Western Sydney University, Penrith, New South Wales, Australia; Universita degli Studi di Perugia, ITALY

## Abstract

Sodium ions (Na^+^) are key regulators of molecular events in many cellular processes, yet the dynamics of this ion remain poorly defined. Developing approaches to identify and characterise Na^+^ microenvironments will enable more detailed elucidation of the mechanisms of signal transduction. Here we report the application of Spectral Phasor analysis to the Na^+^ fluorophore, CoroNa Green, to identify and spatially map spectral emissions that report Na^+^ microenvironments. We use differentiating stem cells where Na^+^ fluxes were reported as an antecedent. Myoblast stem cells were induced to differentiate by serum starvation and then fixed at intervals between 0 and 40-minutes of differentiation prior to addition of CoroNa Green. The fluorescent intensity was insufficient to identify discrete Na^+^ microenvironments. However, using Spectral Phasor analysis we identified spectral shifts in CoroNa Green fluorescence which is related to the Na^+^ microenvironment. Further, spectral-heterogeneity appears to be contingent on the distance of Na^+^ from the nucleus in the early stages of differentiation. Spectral Phasor analysis of CoroNa Green in fixed stem cells demonstrates for the first time that CoroNa Green has unique spectral emissions depending on the nature of the Na^+^ environment in differentiating stem cells. Applying Spectral Phasor analysis to CoroNa Green in live stem cells is likely to further elucidate the role of Na^+^ microenvironments in the differentiation process.

## Introduction

Sodium ions play many important roles in cells. They can propagate electrical signals in excitable cells, induce contractions in muscle [1, 2], regulate intracellular pH levels[3] and cell cycle progression[3], initiate proliferation[4] and differentiation[5]. Blocking Na^+^ influxes through the inhibition of sodium transporters attenuates proliferation, cell cycle regulation, and differentiation [1–5].

One way that cations such as Na^+^ influence the activity of cells is by regulating the activity of proteins. Many proteins are metalloproteins that have their activity regulated via complexation with metal ions [6]. These enzymes can take advantage of cations such as Na^+^ inside cells as a source of chemical potential to facilitate the binding and catalysis of substrates[7]. Sodium ions can also influence the conformation of other macromolecules such as DNA through electrostatic interactions, and consequently may affect gene expression. Since DNA has a negative charge due to the phosphates on the backbone, it requires counter ions for charge balancing. Sodium ions bind electrostatically to within a few angstroms of the DNA surface where they form a cloud of ions which are still free to move along the helix structure [8]. Monovalent ions such as Na^+^ primarily influence the minor-groove structure of DNA by reducing its effective charge. This affects DNA solution properties, stability, and binding interactions with proteins [9]. The role of sodium in gene expression is further supported by the localization[10] and the colocalization[11] of Na^+^ transporters such as Na^+^-Ca^2+^ and Na^+^-K^+^ exchangers in the inner nuclear envelope. These transporters work to regulate the movement of Na^+^, altering local and global concentrations in the nucleus. However, there is a paucity of *in vivo* evidence to demonstrate the involvement of Na^+^ in gene expression.

Characterising the biochemical environment of sodium would enable a greater understanding of its role in the cell. Previous studies of cellular Na^+^ fluxes have relied on fluorescent probes that undergo fluorescence intensity changes upon Na^+^ binding [12]. Sodium-binding benzofuran isophthalate (SBFI) is one fluorophore that has been utilised to study Na^+^ in mammalian cells. This fluorescent molecule is observed to be 20-fold more specific for Na^+^ than for K^+^ [13]. In the presence of K^+^, the dissociation constant (K_d_) is 11.3 mM making it suitable for the detection of small changes in Na^+^ concentration. Upon binding to Na^+^, SBFI’s quantum yield increases causing a narrowing of the excitation peak and a shift in the excitation maximum to shorter wavelengths resulting in a significant change in the ratio of fluorescence excited at 340 nm/380 nm[14]. This property enables ratio-metric fluorescence analysis for the determination of Na^+^ concentrations independent of fluorophore concentration[15]. Since SBFI has a low excitation wavelength in the UV range, the utilisation of this fluorophore necessitates UV lasers or two-photon imaging. The cost and complexity of these methods has been a barrier to widespread usage of this fluorophore[14]. One further disadvantage of the SBFI dye is that it has low cell permeability despite the addition of dispersion agents such as Pluronic F127. This is a disadvantage because long exposure times and high indicator concentrations induce cellular stress causing artefacts[16].

Sodium Green is an alternate fluorophore that has an excitation/emission in the visible light spectrum (507 nm/532 nm) enabling the utilisation of the Argon 488 laser. It also has greater selectivity for Na^+^ ions than SBFI (41-fold versus 18-fold), a K_d_ value of 21 mM in the presence of K^+^, making it suitable for measurements of physiological changes in Na^+^ concentration[14, 17]. However, Sodium Green has been shown to interact with proteins, measured by the attenuation of fluorescence and a shift in the life time in FLIM analysis in the presence of 5% BSA.[18].

CoroNa Green is excitable in the visible light spectrum (ex: 492 nm, em: 516 nm) and exhibits a concentration dependent increase in fluorescence with little spectral shift[14]. CoroNa Green has a 4-fold selectivity towards Na^+^ over K^+^, and unlike Sodium Green has not yet been shown to interact with proteins[14]. It is approximately half the size of Sodium Green (586 g/mol versus 1668 g/mol) which ostensibly assists diffusion of the molecule into the cell[14, 17]. However, in previous studies fluorescence intensity of CoroNa Green has been shown to decline over time, and this has been attributed to the leakage of the fluorescent molecule from the cell due to its relatively low molecular weight[14, 16].

Due to the high K_d_ value (~80 mM), CoroNa Green has been shown[14, 17] to be insensitive to Na^+^ concentrations fluctuations in the range of 0 and 50 mM; however, this larger Kd enables analysis of broader concentration of Na^+^ and larger Na^+^ transients. CoroNa Green has previously been employed to investigate Na^+^ distribution in regenerating amphibian limbs [12] and in following Na^+^ influxes of stimulated neurons [19].

Fluorescence intensity measurements are inherently limited to interrogating the concentration and spatial distribution of Na^+^ and do not enable characterisation and analysis of biochemical changes. Spectroscopy approaches acquire spectral and fluorescence intensity data across a given range of wavelengths[20] and can provide information about the biochemical environment of environmentally sensitive probes. The emission spectra of an environmentally sensitive fluorescent probe are reportedly contingent on many factors including, pH, ion concentration, molecular interactions, and protein binding[21].

Spectral imaging has limitations due to the need for complex analysis algorithms and the requirement for understanding a fluorescent molecule’s spectral profile prior to characterization[15]. However, the Phasor approach to spectral imaging addresses these limitations by removing the need for complex algorithms and prior knowledge of a probe’s spectral characteristics, and instead visually representing the spectral emission in a scatter plot (Phasor plot)[15]. Selection of emission characteristics, (λ_max_ and spectral width), in the phasor plot enables the mapping of these characteristics to the original fluorescence image enabling both regional and cell wide analysis[15, 22]. Spectral Phasor analysis has been successfully utilised to characterise microenvironments in mammalian cells[21], auto-fluorescence in living grass[15], membrane dynamics in root hair cells[23], and lamellar body-like structures in A549 cells[24].

CoroNa Green has been reported to not undergo any wavelength shifts after addition to cells and is not widely considered an environmentally sensitive reporter [14, 17]. However, given the ability of the fluorophore to diffuse through many different cell compartments it is possible that spectral shifts have remained hidden. The main aim of the present study is to determine the ability of Spectral Phasor analysis to detect any spectral shifts. Additionally, given the importance of Na^+^ fluxes in differentiating cells, we sought to determine whether Spectral Phasor analysis in conjunction with CoroNa Green could identify different Na^+^ microenvironments spatially in the cell, and temporally in differentiating stem cells. This will allow a more complete understanding of the biochemical changes occurring due to differentiation of stem cells.

## Method

### Cell culture

Rat (L6) myoblasts (ATCC CRL-1458) were cultured for 48 hours prior to fixation on a 35-mm diameter glass bottom dish (WPI) containing cell growth media (2mL DMEM, supplemented with 10% Foetal Bovine Serum (FBS) and Penicillin/Streptomycin), and maintained in a 37°C, 5% CO_2_ humidified incubator.

### Cell treatment and fixation

Cells were induced to differentiate via serum starvation by replacing complete DMEM (10% FBS) with incomplete DMEM (2% FBS)[25]. Formaldehyde fixation (4%) was performed at 10, 20, 30, and 40-minutes post induction of differentiation. Control cells were also fixed with 4% formaldehyde without serum starvation. Formaldehyde fixation was performed by aspirating the media and adding 1 ml of 4% formaldehyde for 10 minutes. To reduce the loss of bound Na^+^ within the cells, the cells were washed 3 times with 1X phosphate buffer saline (PBS) (1 ml), and then maintained in 500μL of 1X PBS and kept at -80 ^o^ C until confocal and spectral data acquisition.

### Data acquisition

All confocal and spectral data were acquired using the Leica TCSPC 5 Confocal inverted confocal microscope with a HCX PL APO CS 63 x 1.2 water objective paired with an Argon 488 laser. Just prior to imaging, the cells were loaded with CoroNa Green (0.5 μM) and incubated for 3 minutes at room temperature to allow diffusion into the cell. CoroNa Green was excited at 488 nm (2% of maximum laser power) and the emission captured at 516 nm. For all spectral data acquisition, a detection range of 413 nm—728 nm, scan speed of 100 Hz, band width of 9.7 nm, and 32 detection steps was employed to acquire spectral images with a resolution of 256 x 256 at 12 bits.

### Data analysis

Spectral data were loaded into SimFCS 4.0[26], ‘referenced’ and ‘read’. The Phasor plot was calibrated for the acquisition range of 413 nm– 728 nm, and the background was eliminated by using a threshold in the ‘photon counting histogram’ which removes photons attributed to the extracellular space.

The distribution of CoroNa Green’s emissions in whole cells undergoing differentiation (first 40-mintues) was represented in a 3D scatter plot. This was accomplished by utilising cursors (size = 0.005) to select all the emissions in the phasor plot after the removal of background emissions. The number of data points (n) collected for cells at each time course was contingent on the spread of the phasor (the length and breadth of the phasor).

Analysis of specific regions of interests in the spectral image was performed by using the mask function in SimFSC 4.0. The mask function enables isolation of spectral emissions pertaining to the region of interest. These regions were isolated by drawing a circle with a diameter of 1μm in increments of 1μm, corresponding to the extracellular space, cytoplasmic membrane, regions within the cytoplasm, the nucleus and the nucleolus. The isolated spectral emissions in the phasor plot corresponding to these regions were then represented in a scatter plot.

Spectral heat maps which depict areas of the cell that share similar spectral properties were produced by linking 5 cursors (size = 0.045), which were placed on the phasor plot corresponding to 514 nm, to 5 cursors that were placed at 549 nm. While these groups of 5 cursors shared the same λ_max_, they all differed in spectral width. To only make comparisons of the λ_max_, the same colour scheme was used for each linked cursor. Transects from the cytoplasmic membrane into the cytosol and along the nuclear membrane were generated to compare these isolated regions.

## Results

**Fig 1:**
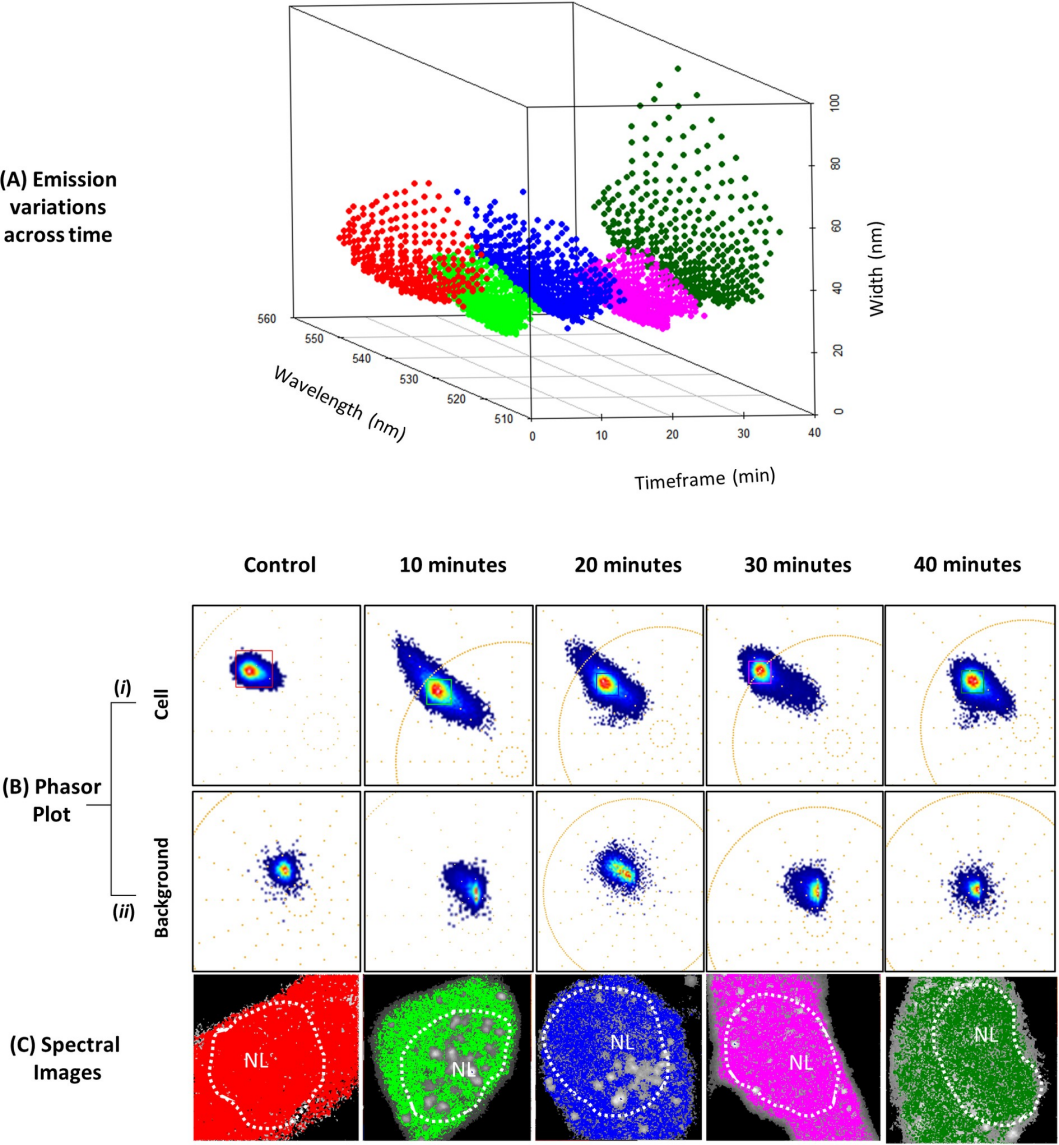
Spectral emission variations as a function of time post serum starvation. (A) 3D scatter plot was generated by utilising cursors (size = 0.005) to select wavelengths and widths in the phasor plot corresponding to each time course (control: n = 274, 10 min: n = 471, 20 min: n = 519, 30 min: n = 439, 40 min: n = 370). The number of data points collected (n) depended on the shape of the phasor; a condensed phasor resulted in lower ‘n’ value, conversely a more spread out phasor resulted in a higher n value. (B-*i*) The phasor shape of emissions corresponding to the cell after the removal of background emissions. (B-*ii*) The phasor shape of emissions corresponding to the background after the removal of the cell emissions. Relative to the cellular emissions, background emissions were apparent near the centre of the plot and exhibited broader widths. (C) A different coloured cursor was utilised to select the most intense region of the phasor plot for each time course, and the emissions were mapped to the original fluorescence images to generate spectral images.

Firstly, we aimed to characterise the spectral properties of CoroNa Green after inducing L6 rat myoblast progenitor cells to differentiate via serum starvation. Intriguingly, the emission spectral shifts (λ_max_ and spectral width) appeared to be contingent on the time post induction of differentiation (Fig 1A). The mean λ_max_ of CoroNa Green did not significantly change and appeared to oscillate around 535 nm throughout the first 40-minutes post induction of differentiation. However, the degree of variation in the λ_max_, as indicated by the standard deviation, shifted from 7.4 nm in control cells to 4.8 at 10 minutes, 6.8 at 20-minutes, 5.8 at 30-minutes, and 8.8 at 40-minutes.

The mean width of emissions experienced a decrease by an average of 12.4 nm in cells 10–30-minutes post serum starvation. The mean width of control cells was 34.7 nm; however, for cells 10–30 minutes post induction of differentiation, the width oscillated around 24 nm but then increased to 37.8 nm at 40-minutes. Coinciding with the decrease in spectral width at 10–30 minutes was also an oscillation in the degree of variance. The standard deviation of emission widths in control cells was 8.2 nm, 5.3 nm for cells at 10-minutes, 7.7 nm at 20-minutes, 5.3 nm at 30-minutes, and 13.9 nm at 40-minutes. These data also make apparent that while the degree of spectral variance was most similar in cells at 40-minutes and control cells, cells at 40-minutes exhibited greater spectral variance. This can be seen in the 3D scatter plot which maps the distribution of CoroNa Green’s emissions within the first 40-minutes of serum starvation and in the shape of the phasor (Fig 1). The scatter plot depicts a higher degree of dispersion in the emission of cells at 40-minutes compared to all other time courses (Fig 1A). When comparing the phasor of cells at 10–30 minutes post serum starvation to control cells, the shape appears to have undergone elongation, indicating a greater degree of spectral width shifts rather than shifts in λ_max_. However, at 40-minutes the phasor not only appears to have maintained a relatively elongated shape, but it also seems to have broadened, which indicates a greater degree of both width and λ_max_ shifts.

**Fig 2:**
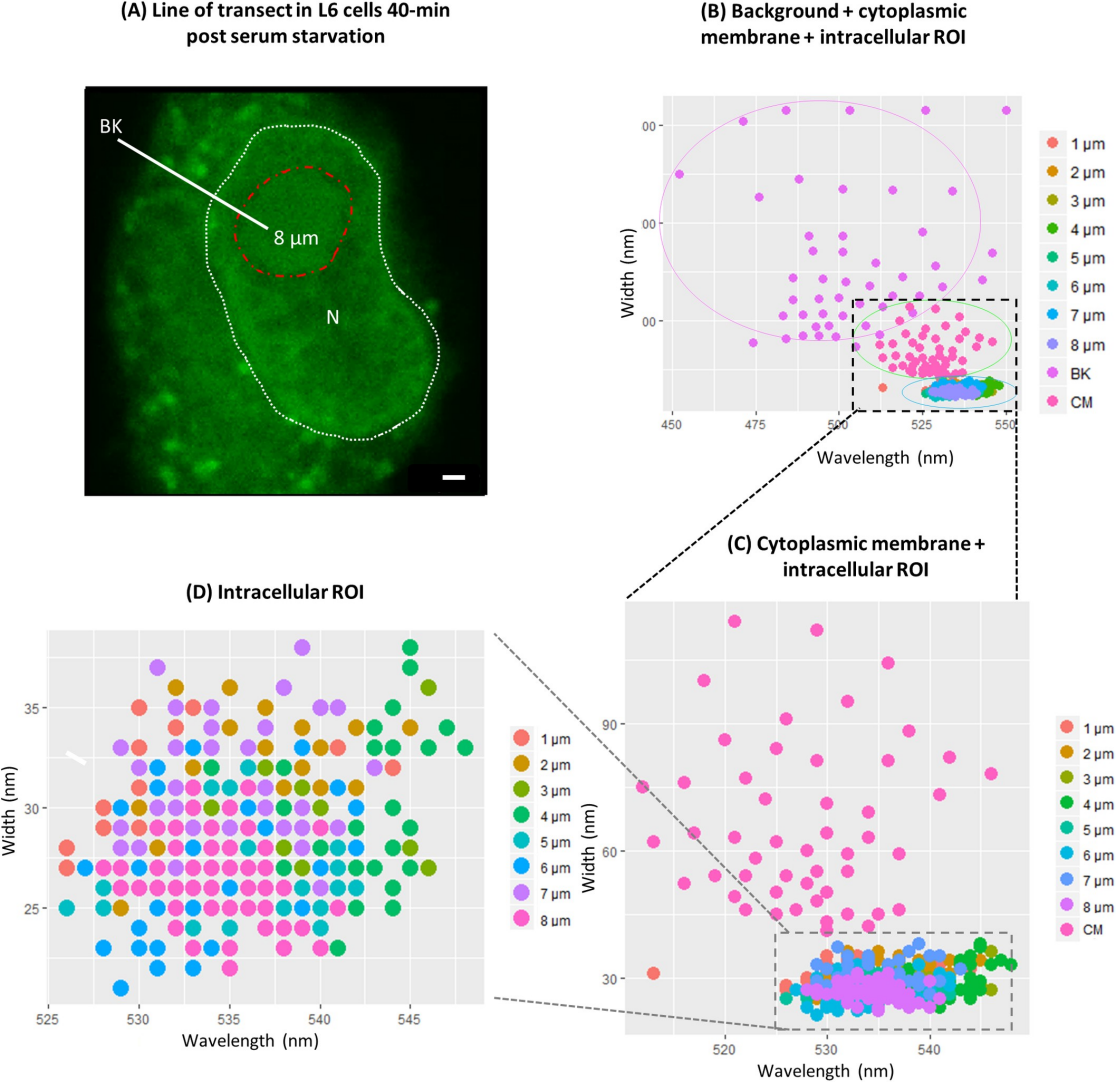
Spectral variation with respect to distance from cytoplasmic membrane. Regions of the spectral image along the transect line (A) at 1 μm increments were isolated and 50 cursors (size = 0.005) were utilised to select emissions on the phasor plot corresponding to the selected regions of interest. (B) The variance of emissions appeared greater in the background (indicated by the largest circle) than the cytoplasmic membrane which was more variable than emissions found within the cell. (C) Removing the background emissions made apparent the difference in the degree of variability between the cytoplasmic membrane emissions compared to regions within the cell. (D) Within the population of emissions found 1–8 μm within the cell, each region’s emissions appeared to converge in non-overlapping parts of the spectrum; however, these λ_max_ did share similar spectral widths. Note that the colour code for each scatter plot changes for each cell region from (B) to (D) as the background and cytoplasmic membrane emission are removed. White dotted line represents the nucleus and the red dotted line represents the nucleolus.

To determine the sensitivity of Spectral Phasor analysis to spatially contingent spectral shifts, the distribution of emissions across a line of transect from the extracellular space to the nucleolus was compared (Fig 2). The greatest degree of variation in spectral emissions was apparent outside of the cell (background) corresponding to emissions found near the centre of the phasor plot (Fig 1B), followed by the cytoplasmic membrane region, and then regions corresponding to the cytosol and the nucleus of the cell.

**Fig 3:**
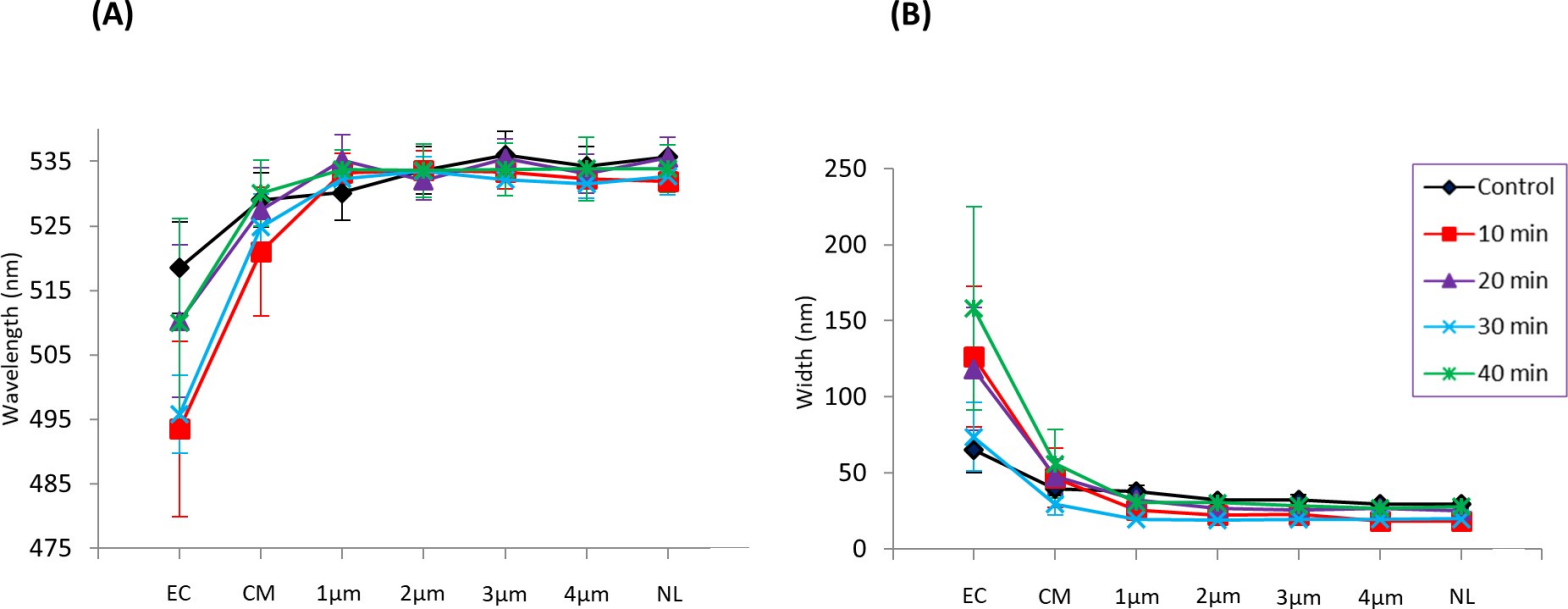
CoroNa Green spectral variations with respect to increasing distance from the cytoplasmic membrane. A line of transect was drawn from 1 μm outside of the cell to the nucleolus. The location of the transect line was varied to ensure equidistance from the extracellular space to the nucleolus in all the time courses. Spectral emissions were isolated by selecting regions along the line of transect at 1 μm increments. Graphs were created by utilising small cursors (size = 0.005) to select λ_max_ and widths on the phasor plot corresponding to the cellular region. (A) Depicts smaller λ_max_ values in the extracellular space and near the cytoplasmic region compared to intracellular regions. (B) Depicts a decrease in the width of emissions with respect to a decrease in the distance from the nucleolus. EC = 1 μm outside of cell, CM = cytoplasmic membrane, and NL = nucleolus.

Analysis of spatially contingent λ_max_ fluctuations revealed variations that were similar across all time course (Fig 3A). CoroNa in regions within the cell (control– 40 minutes post serum starvation) exhibited larger wavelengths with a lower degree of variation compared to the cytoplasmic membrane region and the extracellular space. The width of emissions appeared largest in the vicinity of the cytoplasmic membrane and the extracellular space relative to intracellular regions (Fig 3B).

**Fig 4:**
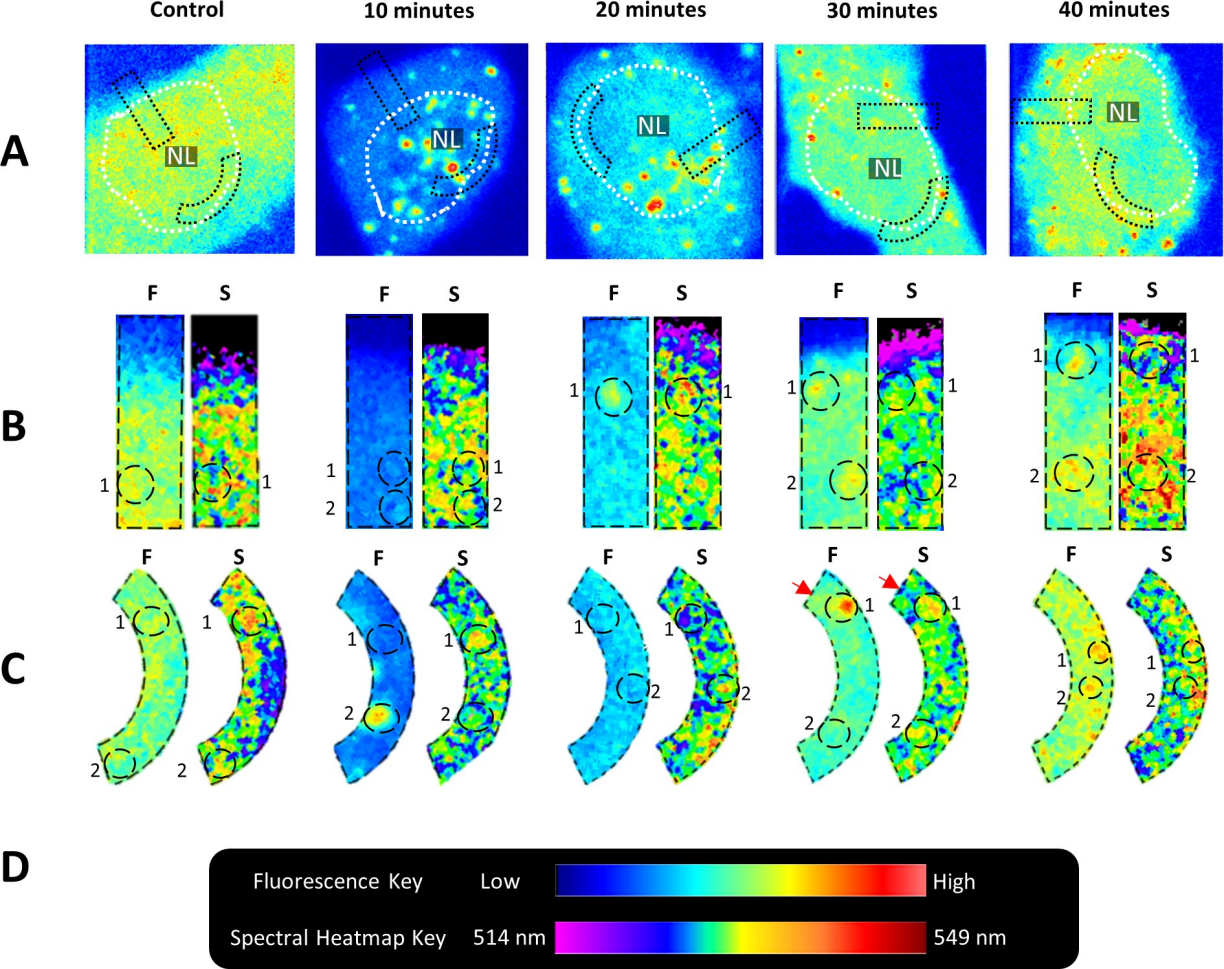
Sodium microenvironment heat map in myoblast progenitor cells induced to differentiate. (A) Fluorescence images (photons collected in the range of 413–728 nm) depicting lower levels of fluorescence in the nucleus and cytoplasm of cells at 10 and 20 minutes post induction of differentiation relative to control cells and cells at 30 and 40 minutes. White border indicates nucleus and NL = Nucleolus. Regions of interest were isolated, and fluorescence intensity images and spectral heat maps of (B) a transect from the cytoplasmic membrane into the cytosol and (C) the nuclear membrane region, were produced. ‘F’ indicates fluorescence intensity image, while ‘S’ indicates spectral heat map. The heat map depicts areas within the region of interests that share similar spectral properties via pseudo colouring (B and C–‘S’). Dotted circles within isolated areas provide a comparison of specific fluorescence intensities and spectral heat map ‘microenvironments’. Red arrow indicates region with similar fluorescence intensity but different spectral properties.

Fluorescence intensity of CoroNa Green appeared lowest in the vicinity of the cytoplasmic membrane (Fig 4A). Transects from the extracellular space to the nucleus were taken to compare the distribution of emissions with reference to the fluorescence intensity (Fig 4B and 4C). In the transect (Fig 4B) and the nuclear membrane region (Fig 4C) of both control cells and cells induced to differentiate, the same Na^+^ concentrations did not appear to correlate with specific ‘microenvironments’ in the heat map. For instance, in cells 40-minutes post induction of differentiation, selected regions outlined by dotted circle 1 indicates the colocalisation of higher fluorescence intensity with lower wavelengths (blue colour in spectral heat map) in the vicinity of the cytoplasmic membrane (Fig 4B, 40-minutes). In contrast, in the selected region outlined by dotted circle 2, the higher fluorescence intensity appeared to colocalise with higher wavelengths (orange/red colour) in the heat map.

This phenomenon was also apparent when observing the nuclear membrane (Fig 4C, 40-min); the selected region (dotted circle 1) exhibited mainly higher fluorescence (indicated by dark orange and yellow colours). However, this region (dotted circle 1) had distinct sub-microenvironments as indicated by the orange and yellow colours (north and south sections within the circle–assuming top of the page as north), and the green and blue (towards the east and west regions) in the spectral heat map. Similarly, in the dotted circle 2 (Fig 4C, 40-min), the same fluorescence intensity (yellow/orange) correlated with both the cyan and green regions in the heat map. Additionally, the high fluorescence areas also correlated with the lower wavelength (yellow) microenvironments in the heat map (see dotted circle and red arrows in Fig 4C, 30-min).

**Fig 5:**
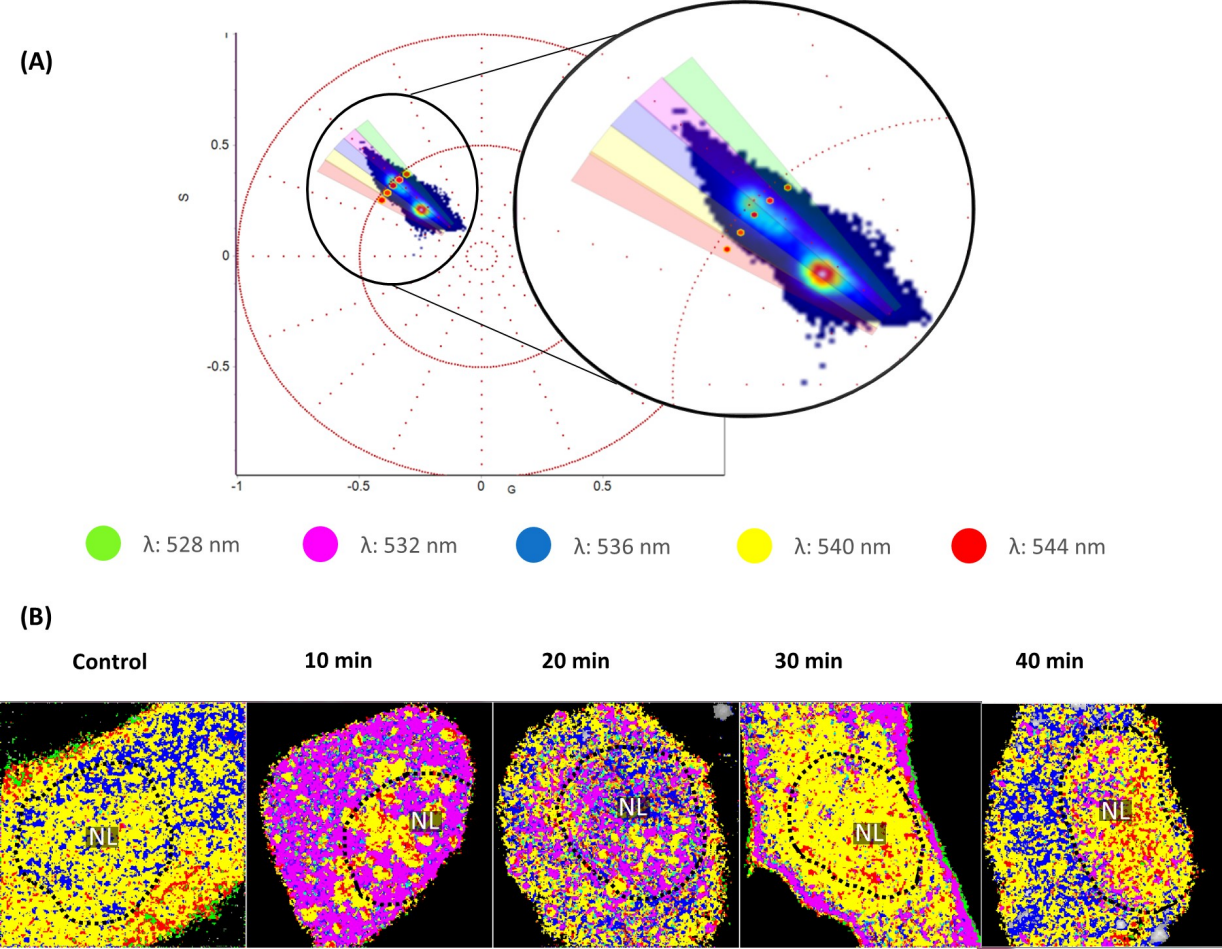
Spectral analysis of control and serum starved myoblast progenitor cells. (A) Phasor plot depicting the phasor position of control and serum starved cells in the second quadrant of first harmonic. Four cone shaped cursors were utilised to select four distinct λ_max_ values irrespective of the spectral width. (B) Spectral images were produced by remapping the selected emissions in the (A) phasor plot to the original fluorescence images. Notably, the 532, 536, 540 and 544 nm emissions were apparent in both the nucleus and cytoplasm of cells throughout the first 40-minutes post serum starvation.

Selection of four distinct λ_max_ values, irrespective of spectral width was mapped back to the original fluorescence image. It was apparent that λ_max_ values which were in the cytosol were also apparent in the nucleus. This was the case for the 532 nm, 536 nm, 540 nm and 544 nm emissions (Fig 5B).

**Fig 6:**
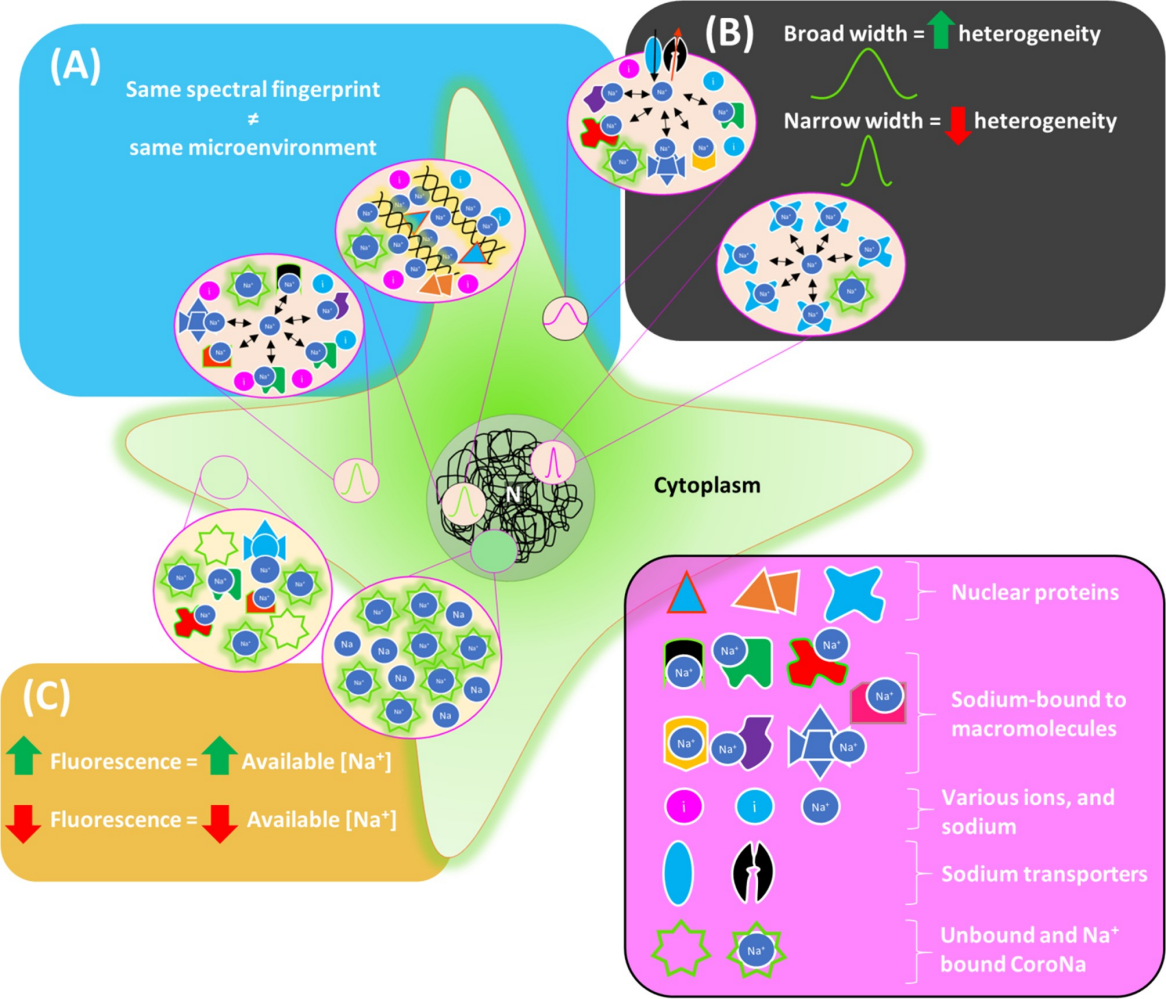
Schematic summary of results. (A) Depicts two distinct microenvironments with overlapping emissions (refer to Fig 5). (B) Spectral breadth decreasing with decreasing level of microenvironment heterogeneity (refer to Figs 3 and 4B). (C) The fluorescence of CoroNa Green indicates available Na^+^ rather than actual concentration in a given cellular region (refer to Fig 1). N = Nucleus.

Based on the findings of the current study, the model that appears to be developing has been summarised schematically in Fig 6.

## Discussion

In this study we sought to determine whether Spectral Phasor analysis could recover Na^+^ microenvironment information by detecting spectral shifts that were temporally and spatially contingent in fixed rat myoblast progenitor cells. Comparison of spectral emissions in control cells and cells induced to differentiate within the first 40-minutes revealed spectral shifts that appeared to be time dependent. We observed a narrowing of spectral width within the first 30-minutes post serum starvation, followed by broadening at 40-minutes (Fig 1). The breadth of the emission reflects the heterogeneity of the processes occurring in that molecular environment. This is because an emission with a broad width is comprised of multiple wavelengths exhibiting varied intensities[20]. The individual wavelengths are indicative of specific molecular environments or biochemical processes. The narrowing of emission widths within the first 30-minutes suggests a decrease in microenvironment heterogeneity. This indicates that when the serum is reduced to initiate differentiation, there is a concerted biochemical response in rat myoblast progenitor cells. This is further supported by the observed reduction in wavelength variance, particularly at 10 and 30-minutes post serum starvation.

The biochemical response of rat myoblast cells to serum starvation appears to directly involve Na^+^. This is supported by fluorescence intensity measurements which depict the lowest degree of fluorescence at 10 and 20-minutes (Fig 4). Since CoroNa Green detects Na^+^ by trapping the ion in the crown ether of the molecule[14], the fluorescence intensity does not simply indicate the concentration of Na^+^ in a particular region of the cell. Instead, a lack of fluorescence may suggest the unavailability of Na^+^ because of competitive binding to other macromolecules. If serum starvation activates biochemical pathways that involve enzymes which utilise Na^+^ for its activation, then we would expect to see a decrease in fluorescence intensity, as seen in Fig 4A. Intriguingly, the fluorescence intensity appears to have decreased both in the cytoplasm and in the nucleus, suggesting Na^+^ utilization in both these regions of the cell (Fig 4A). The recovery of fluorescence intensity at 40-minutes appears to coincide with the broadening of spectral width and increased variance in the λ_max._ This may suggest the presence of a more heterogeneous biochemical landscape that does not directly utilise Na^+^. These data appear to agree with previous studies which report Na^+^ influxes as an early antecedent to downstream processes such as proliferation[4] and differentiation[27].

To determine whether the Spectral Phasor approach is capable of detecting shifts that are spatially contingent, we isolated emissions corresponding to regions of interest along a line of transect (Fig 2). These regions of interest included the extracellular space (background), the cytoplasmic membrane area, regions within the cytoplasm, the nucleus and nucleolus. With respect to regions within the cell, we expected the area in the vicinity of the cytoplasmic membrane to exhibit the largest degree of variation. This is because Na^+^ in this region may well be fluxing in and out of the cell[28, 29], dynamically binding to proteins and other macromolecules[30], which ultimately increases the microenvironment heterogeneity. The results in Figs 2 and 3 which depicts the cytoplasmic membrane microenvironment as the most heterogeneous and the nuclear microenvironment as the least, agrees with our expectations. These data demonstrate that Spectral Phasor analysis can detect difference in microenvironment heterogeneity in different regions of cells.

We next investigated the relationship between CoroNa Green’s fluorescence intensity and λ_max_. Our results show that even if two regions of the cell exhibit the same fluorescence intensity, it does not follow that always share the same spectral properties, i.e. the same microenvironment (Fig 4B and 4C). This was apparent when comparing regions which transect the cytoplasmic membrane as well as the nuclear membrane region (compare dotted circles in the fluorescence images and spectral heat maps of Fig 4C, 40-min). These data indicate that despite similar or the same Na^+^ concentration in these regions, it is likely that different molecular interactions and biochemical processes take place[1, 11]. It was also observed that regions which differed in fluorescence intensity often exhibited similar spectral properties (compare dotted circles in Fig 4C, 30-min), suggesting that despite disparate Na^+^ concentrations, similar biochemical processes are taking place. A caveat that is worth mentioning is that even if two regions of the cell exhibit similar or even the same spectral fingerprint, it does not necessarily follow that these environments are biochemically identical. For instance, in Fig 5 it can be seen that emissions exhibited in the cytosol are also apparent in the nucleus of cells. Considering how different the nuclear environment is compared to the cytosol, one cannot conclude that these microenvironments are identical despite the similarity in the emissions spectra. Similar to other spectroscopy techniques such as infrared spectroscopy[31] in which distinct chemical groups overlap and share spectral properties, it is possible, if not expected, that such a phenomenon also exists with Spectral phasor analysis.

## Conclusion

In this study we found that Spectral Phasor analysis can be used to detect emission changes of CoroNa Green both temporally in cells induced to differentiate and spatially in different regions of the cell. This provides evidence for the environmental sensitivity of this probe when used in conjunction with Spectral Phasor analysis. This approach shows that the largest shifts in Na^+^ microenvironments occur within the first 30-minutes of differentiation. Furthermore, we report the heterogeneity of microenvironments was highest near the cytoplasmic membrane, and lowest in the nucleus reflecting the variability of biological processes near the cytoplasmic membrane compared to the nucleus (Fig 6). This knowledge provides a deeper understanding of Na^+^ microenvironments in myoblast progenitor cells. Unlike other biophysical approaches[12, 19] which only elucidate the spatial distribution of Na^+^, SPA when applied to Na^+^ specific fluorophores such as CoroNa, provides further information about the dynamic biochemical and molecular biological changes in sodium ion’s local environment.

## References

[1] Aronsen J, Swift F, Sejersted O. Cardiac sodium transport and excitation–contraction coupling. J Mol Cell Cardiol. 2013;61:11–9. 10.1016/j.yjmcc.2013.06.003 23774049

[2] Verdonck F, Mubagwa K, Sipido KR. [Na+] in the subsarcolemmal ‘fuzzy’space and modulation of [Ca 2+] i and contraction in cardiac myocytes. Cell Calcium. 2004;35(6):603–12. 10.1016/j.ceca.2004.01.014 15110150

[3] Putney LK, Barber DL. Na-H exchange-dependent increase in intracellular pH times G2/M entry and transition. J Biol Chem. 2003;278(45):44645–9. 10.1074/jbc.M308099200 12947095

[4] Koch K, Leffert H. Increased sodium ion influx is necessary to initiate rat hepatocyte proliferation. Cell. 1979;18(1):153–63. 509519 10.1016/0092-8674(79)90364-7

[5] Wang H, Singh D, Fliegel L. The Na+/H+ antiporter potentiates growth and retinoic acid-induced differentiation of P19 embryonal carcinoma cells. J Biol Chem. 1997;272(42):26545–9. 9334233 10.1074/jbc.272.42.26545

[6] Yannone SM, Hartung S, Menon AL, Adams MW, Tainer JA. Metals in biology: defining metalloproteomes. Curr Opin Biotechnol. 2012;23(1):89–95. 10.1016/j.copbio.2011.11.005 22138493 PMC3273585

[7] Gohara DW, Di Cera E. Molecular mechanisms of enzyme activation by monovalent cations. J Biol Chem. 2016;291(40):20840–8. 10.1074/jbc.R116.737833 27462078 PMC5076497

[8] Blackburn GM. Nucleic acids in chemistry and biology: Royal Society of Chemistry; 2006.

[9] Kettani A, Bouaziz S, Gorin A, Zhao H, Jones RA, Patel DJ. Solution structure of a Na cation stabilized DNA quadruplex containing G· G· G· G and G· C· G· C tetrads formed by GGGC repeats observed in adeno-associated viral DNA. J Mol Biol. 1998;282(3):619–36. 10.1006/jmbi.1998.2030 9737926

[10] Garner M. Na, K-ATPase in the nuclear envelope regulates Na+: K+ gradients in hepatocyte nuclei. J Membr Biol. 2002;187(2):97–115. 10.1007/s00232-001-0155-5 12029368

[11] Galva C, Artigas P, Gatto C. Nuclear Na+/K+-ATPase plays an active role in nucleoplasmic Ca2+ homeostasis. J Cell Sci. 2012;125(24):6137–47. 23077175 10.1242/jcs.114959 PMC3585523

[12] Tseng A-S, Beane WS, Lemire JM, Masi A, Levin M. Induction of vertebrate regeneration by a transient sodium current. J Neurosci. 2010;30(39):13192–200. 10.1523/JNEUROSCI.3315-10.2010 20881138 PMC2965411

[13] Minta A, Tsien RY. Fluorescent indicators for cytosolic sodium. J Biol Chem. 1989;264(32):19449–57. 2808435

[14] Meier SD, Kovalchuk Y, Rose CR. Properties of the new fluorescent Na+ indicator CoroNa Green: comparison with SBFI and confocal Na+ imaging. J Neurosci Methods. 2006;155(2):251–9. 10.1016/j.jneumeth.2006.01.009 16488020

[15] Fereidouni F, Bader AN, Gerritsen HC. Spectral phasor analysis allows rapid and reliable unmixing of fluorescence microscopy spectral images. Opt Express. 2012;20(12):12729–41. 10.1364/OE.20.012729 22714302

[16] Iamshanova O, Mariot P, Lehen’Kyi VY, Prevarskaya N. Comparison of fluorescence probes for intracellular sodium imaging in prostate cancer cell lines. Eur Biophys J. 2016;45(7):765–77. 10.1007/s00249-016-1173-7 27660079 PMC5045488

[17] Schreiner A, Rose C. Quantitative imaging of intracellular so dium. Current Microscopy Contributions to Advances in Science and Technology (Méndez-Vilas A, ed). 2012:119–29.

[18] Despa S, Vecer J, Steels P, Ameloot M. Fluorescence lifetime microscopy of the Na+ indicator Sodium Green in HeLa cells. Anal Biochem. 2000;281(2):159–75. 10.1006/abio.2000.4560 10870831

[19] Tyler WJ, Tufail Y, Finsterwald M, Tauchmann ML, Olson EJ, Majestic C. Remote excitation of neuronal circuits using low-intensity, low-frequency ultrasound. PLoS One. 2008;3(10):e3511 10.1371/journal.pone.0003511 18958151 PMC2568804

[20] Garini Y, Young IT, McNamara G. Spectral imaging: principles and applications. Cytometry Part A. 2006;69(8):735–47. 10.1002/cyto.a.20311 16969819

[21] Andrews LM, Jones MR, Digman MA, Gratton E. Spectral phasor analysis of Pyronin Y labeled RNA microenvironments in living cells. Biomed Opt Express. 2013;4(1):171–7. 10.1364/BOE.4.000171 23304656 PMC3539199

[22] CutraleF, SalihA, GrattonE. Spectral phasor approach for fingerprinting of photo-activatable fluorescent proteins Dronpa, Kaede and KikGR. Method Appl Fluoresc. 2013;1(3):035001.10.1088/2050-6120/1/3/03500129148446

[23] Sena F, Sotelo-Silveira M, Astrada S, Botella MA, Malacrida L, Borsani O. Spectral phasor analysis reveals altered membrane order and function of root hair cells in Arabidopsis dry2/sqe1-5 drought hypersensitive mutant. Plant Physiol Biochem. 2017;119:224–31. 10.1016/j.plaphy.2017.08.017 28910707

[24] Malacrida L, Astrada S, Briva A, Bollati-Fogolín M, Gratton E, Bagatolli LA. Spectral phasor analysis of LAURDAN fluorescence in live A549 lung cells to study the hydration and time evolution of intracellular lamellar body-like structures. Biochimica et Biophysica Acta (BBA)-Biomembranes. 2016;1858(11):2625–35. 27480804 10.1016/j.bbamem.2016.07.017 PMC5045802

[25] Yaffe D, Saxel O. A myogenic cell line with altered serum requirements for differentiation. Differentiation. 1977;7(1–3):159–66. 558123 10.1111/j.1432-0436.1977.tb01507.x

[26] Gratton E. Globals Software for Spectroscopy and Images [Available from: http://www.lfd.uci.edu/globals/.

[27] Ladoux A, Miglierina R, Krawice I, Cragoe EJ, Abita JP, Frelin C. Single‐cell analysis of the intracellular pH and its regulation during the monocytic differentiation of U937 human leukemic cells. Eur J Biochem. 1988;175(3):455–60. 3165863 10.1111/j.1432-1033.1988.tb14216.x

[28] Leblanc N, Hume JR. Sodium current-induced release of calcium from cardiac sarcoplasmic reticulum. Science. 1990;248(4953):372–7. 2158146 10.1126/science.2158146

[29] Lederer WJ, Niggli E, Hadley RW. Sodium-calcium exchange in excitable cells: fuzzy space. Science. 1990;248(4953):283–4. 2326638 10.1126/science.2326638

[30] Page MJ, Di Cera E. Role of Na+ and K+ in enzyme function. Physiol Rev. 2006;86(4):1049–92. 10.1152/physrev.00008.2006 17015484

[31] Stuart B. Infrared spectroscopy: Wiley Online Library; 2005.

